# Impact of exercise dosages based on American College of Sports Medicine recommendations on lipid metabolism in patients after PCI: a systematic review and meta-analysis of randomized controlled trials

**DOI:** 10.1186/s12944-024-02210-0

**Published:** 2024-07-24

**Authors:** Qing Wen, Xiao-Rong Mao, Juan Wen, Xiao-Juan Yang, Juan Chen, Hu-Kui Han, Xiao-Li Tang, Qun-Hua Ma

**Affiliations:** 1 Department of Cardiology, School of Medicine, Sichuan Provincial People’s Hospital, University of Electronic Science and Technology of China, Chengdu, China; 2 Department of Nursing, School of Medicine, University of Electronic Science and Technology of China, Chengdu, China; 3 General Ward 2, Sichuan Cancer Hospital, Chengdu, China; 4 Otorhinolaryngology, School of Medicine, Sichuan Provincial People’s Hospital, University of Electronic Science and Technology of China, Chengdu, China; 5 Burn Unit, School of Medicine, Sichuan Provincial People’s Hospital, University of Electronic Science and Technology of China, Chengdu, China; 6 Intensive Care Unit, School of Medicine, Sichuan Provincial People’s Hospital, University of Electronic Science and Technology of China, Chengdu, China

**Keywords:** Exercise dosage, Exercise intervention, Coronary heart disease, Lipid metabolism, Exercise dosage, Percutaneous coronary intervention

## Abstract

**Background:**

The impact of exercise dosages based on American College of Sports Medicine(ACSM) recommendations on lipid metabolism in patients after PCI remains unclear. This study conducted a meta-analysis of reported exercise dosages from the literature to address this knowledge gap.

**Methods:**

A comprehensive search of databases was conducted to identify eligible randomized controlled studies of exercise interventions in patients after PCI, following the Preferred Reporting Items for Systematic Reviews and Meta-Analyses (PRISMA) statement. Based on the recommended exercise dosages from ACSM for patients with coronary heart disease, exercise doses in the literature that met the inclusion criteria were categorized into groups that were highly compliant with ACSM recommendations and those with low or uncertain ACSM recommendations. The topic was the effect of exercise dose on lipid metabolism in post-PCI patients. This was assessed using standardized mean difference (SMD) and 95% confidence intervals (95% CI) for changes in triglycerides, total cholesterol, LDL, and HDL.

**Results:**

This systematic review included 10 randomized controlled studies. The subgroup analysis revealed statistically significant differences in the high compliance with ACSM recommendations group for triglycerides [SMD=-0.33 (95% *CI* -0.62, -0.05)], total cholesterol [SMD=-0.55 (95% *CI* -0.97, -0.13)], low-density lipoprotein [SMD=-0.31 (95% *CI* -0.49, -0.13)], high-density lipoprotein [SMD = 0.23 (95% *CI* 0.01, 0.46)], and body mass index [SMD=-0.52 (95% *CI* -0.87, -0.17)]. Compared to the low or uncertain compliance with ACSM recommendations group, the high compliance group exhibited significant differences in improving TC levels (-0.55_(H)_ vs. -0.46_(L)_), HDL levels (0.23_(H)_ vs. 0.22_(L)_), and BMI (-0.52_(H)_ vs. -0.34_(L)_).

**Conclusions:**

This study supports that high compliance with ACSM-recommended exercise dosages has significant impacts on improving TC levels, HDL levels, and BMI. However, no advantage was observed for TG or LDL levels.

**Supplementary Information:**

The online version contains supplementary material available at 10.1186/s12944-024-02210-0.

## Introduction

Coronary heart disease (CHD) has garnered widespread global attention due to its high morbidity and mortality rates [1]. With approximately 15 million deaths attributed to CHD annually, CHD remains the leading cause of death among various diseases. Percutaneous coronary intervention (PCI), characterized by its high safety, minimal invasiveness, and rapid recovery, has emerged as a crucial method for myocardial reperfusion in CHD patients [2]. Nevertheless, patients remain at risk of in-stent restenosis following the procedure [3]. Although the exact mechanisms underlying ISR have not been fully elucidated, research suggests that various factors, including exercise [4], lipid metabolism disorders [5], and stent diameter and type, may contribute to in-stent restenosis occurrence [6].

Lipid metabolism disorders, as pathological conditions, can damage vascular endothelial cells, thereby affecting the effectiveness of revascularization and increasing the risk of major adverse cardiovascular events (MACEs) in PCI patients [7]. Several indicators are typically monitored to assess the lipid metabolism status: total cholesterol (TC), triglycerides (TG), low-density lipoprotein cholesterol (LDL), and high-density lipoprotein cholesterol (HDL).Serum TC、TG and LDL are also associated with the formation of new atherosclerotic plaques in the stent and that the ratio of serum triglycerides to LDL/HDL is an independent predictor of the development of atherosclerosis [8–10]. Therefore, lipid-lowering therapy is considered an essential strategy for reducing recurrent vascular events in PCI patients [11–14].

Previous studies have explored the role of physical activity as a nonpharmacologic intervention for cardiovascular disease in modulating lipid profiles. A study concluded that prolonged exercise is related to improved TC, LDL, TG, and HDL levels [15]. However, the effectiveness of exercise depends not only on the specific type of exercise but also on the degree of exercise stimulation. Typically, the concept of the exercise Dosage is utilized to quantify this stimulus level [16]. Studying the Dosage‒response relationship between exercise and executive function can reveal differences in the impacts of various exercise modalities, durations, and intensities on patients’ executive function [17].

Currently, the impact of exercise on lipid metabolism remains incompletely understood [18, 19], and contradictory results have been reported regarding the relationship between the exercise Dosage and lipid metabolism. Some studies suggest that the exercise Dosage is effective at modifying TC, TG, HDL, and TG levels [20, 21]. However, others have found that the exercise Dosage has minimal impacts on TC and LDL levels [22]. Given the crucial role of the exercise Dosage in treatment, it is an indispensable component of any exercise therapy. A deeper understanding of the relationship between the Dosage and treatment outcomes is crucial for enhancing patients’ health benefits. Nevertheless, the lack of formal statistical comparisons of exercise dosages limits the conclusions that can be drawn. Based on the aforementioned considerations, this study used a meta-analysis method to determine the effect of the exercise Dosage on lipid metabolism in patients undergoing PCI.

The ACSM has developed exercise prescriptions for healthy adults that include recommended dosages of aerobic, resistance, and flexibility exercise for PCI patients [23], which are shown in Table 1.In accordance with the ACSM guidelines, the present systematic evaluation was designed to compare the effects of high adherence to exercise dosages versus those of low or indeterminate adherence to exercise dosages on lipid metabolism in patients postoperating for PCI.

## Methods

This study was conducted in compliance with the Preferred Reporting Items for Systematic Evaluation and Meta-Analysis (PRISMA) guidelines and is registered in PROSPERO (CRD42024520086).

### Search strategy

The researchers searched the PubMed, Embase, Web of Science, and Cochrane English databases. The search timeframe encompassed the inception of these databases up to January 2024. The following search terms were used: (“percutaneous coronary intervention” or “PCI” or “percutaneous coronary revascularization”) AND (“exercise” or “physical activity” or “physical training”). The detailed search strategies are provided in Appendix 1.

### Study selection criteria

The inclusion criteria were as follows: (a) studies were randomized controlled trials; (b) participants were patients who had undergone PCI; (c) intervention measures: the experimental group received any type of exercise, while the control group received no exercise intervention; and (d) given the association between a higher body mass index (BMI) and unfavorable lipid levels, as well as the role of increased adipose tissue in disrupting lipid metabolism [24], the primary outcomes measured in this study included TG levels, TC levels, HDL levels, LDL levels, and BMI.

The exclusion criteria were as follows: (a) the intervention or control group included other surgical or pharmacological interventions as part of the study; (b) studies with ambiguous outcome measures, missing data, and unobtainable data through contact with the authors; and (c) duplicate publications, conference abstracts, and articles without full-text access.

Two researchers independently reviewed the literature for inclusion and exclusion criteria, verified the results, and resolved any discrepancies through discussion or consultation with a third researcher.

### Data extraction

The data were extracted from the included studies by two authors independently. The primary outcomes were changes in TG, TC, HDL, and LDL levels, while the secondary outcome was BMI. The relevant data, including the first author, year of publication, country, sample size, age, intervention frequency, exercise intensity, duration, and type of exercise, were extracted from the Excel records. If the data were presented graphically, Engauge Digitizer 11.3 software was used to extract the numerical values. In cases where studies reported multiple follow-up results, only the longest follow-up data were extracted.

The exercise dosage was assessed according to the recommendations of the ACSM for the development and maintenance of heart, lung, muscular, skeletal, and neurological capabilities in individuals with CHD [23]. Two authors independently assessed the exercise intervention measures to evaluate compliance with the recommended exercise dosage (Table 1). Disagreements were resolved through deliberation or consultation with a third researcher. The scoring system ranged from 0 to 2 for each exercise metric, where 2 indicated full compliance with the standards, 1 indicated uncertain compliance, and 0 indicated noncompliance. According to this scoring system, the percentage of studies that complied with the ACSM-recommended exercise dosage was calculated. A high level is defined as ≥ 70% compliance with ACSM recommendations, and a low or uncertain level of compliance is defined as < 70% compliance with ACSM recommendations.

**Table 1:** ACSM exercise recommendations for PCI patients

Exercise dosage	Cardiorespiratory exercise	Resistance exercise	Flexibility exercise
Frequency	3 days per week	2–3 days per week	More efective on ≥ 2–3 days per week, daily
Intensity/workload	Moderate intensity;50–60%VO^2^R; 64–76%HRmax; RPE of12–13 on a 6–20 scale	Start with 40–50% 1RM, more capable with 60–70% 1RM	Stretch until you feel your muscles being pulled tight or a slight discomfort
Duration	Continuous or cumulative 30 min	Starting with one set of 8–12 repetitions, increase to two sets after about 2 weeks. Perform no more than 8–10 exercises per session.	Static stretching held for 10–30 s, repeated 2–4 times.

HRmax, maximal HR; VO2R, oxygen uptake reserve; RPE, rating of perceived exertion; 1RM, one repetition maximum

### Quality evaluation

The quality of the research methods used was assessed by two sets of authors using the quality assessment criteria recommended by the Cochrane Collaboration [25] for randomized controlled trials that met the inclusion criteria. The Cochrane risk of bias tool (Rob 2) [26], a revised version of the Cochrane tool, was used for this purpose. The evaluation encompassed six domains: random sequence generation, allocation concealment, blinding of participants and personnel, blinding of outcome assessment, incomplete outcome data, and selective reporting [27]. Disagreements were resolved through deliberation or consultation with a third researcher.

### Statistical analysis

The statistical analysis was conducted using RevMan 5.4.1, with the standard mean difference (SMD) employed as the effect indicator. The Higgins *I*^*2*^ statistic was utilized to assess the statistical heterogeneity of the studies [28]. According to the principles for the selection of fixed-effects and random-effects models [29], the fixed-effects model was considered more applicable when the treatment effects were the same across studies and heterogeneity was small (*I²*values < 50% and *P* ≥ 0.1). If there were differences in treatment effects and high heterogeneity (*I²*≥ 50% and *P* < 0.1), a random effects model was used. In the statistical analysis, the studies were categorized into two groups: a group with high adherence to ACSM recommendations and a group with low or uncertain adherence. A descriptive analysis was used to identify sources of heterogeneity, and Begg’s test, and Egger’s test were employed to evaluate publication bias, with *P* < 0.05 considered to indicate statistical significance. In addition, sensitivity analyses were conducted to assess the robustness of the findings by excluding each study.

## Results

### Study selection

**Fig. 1:**
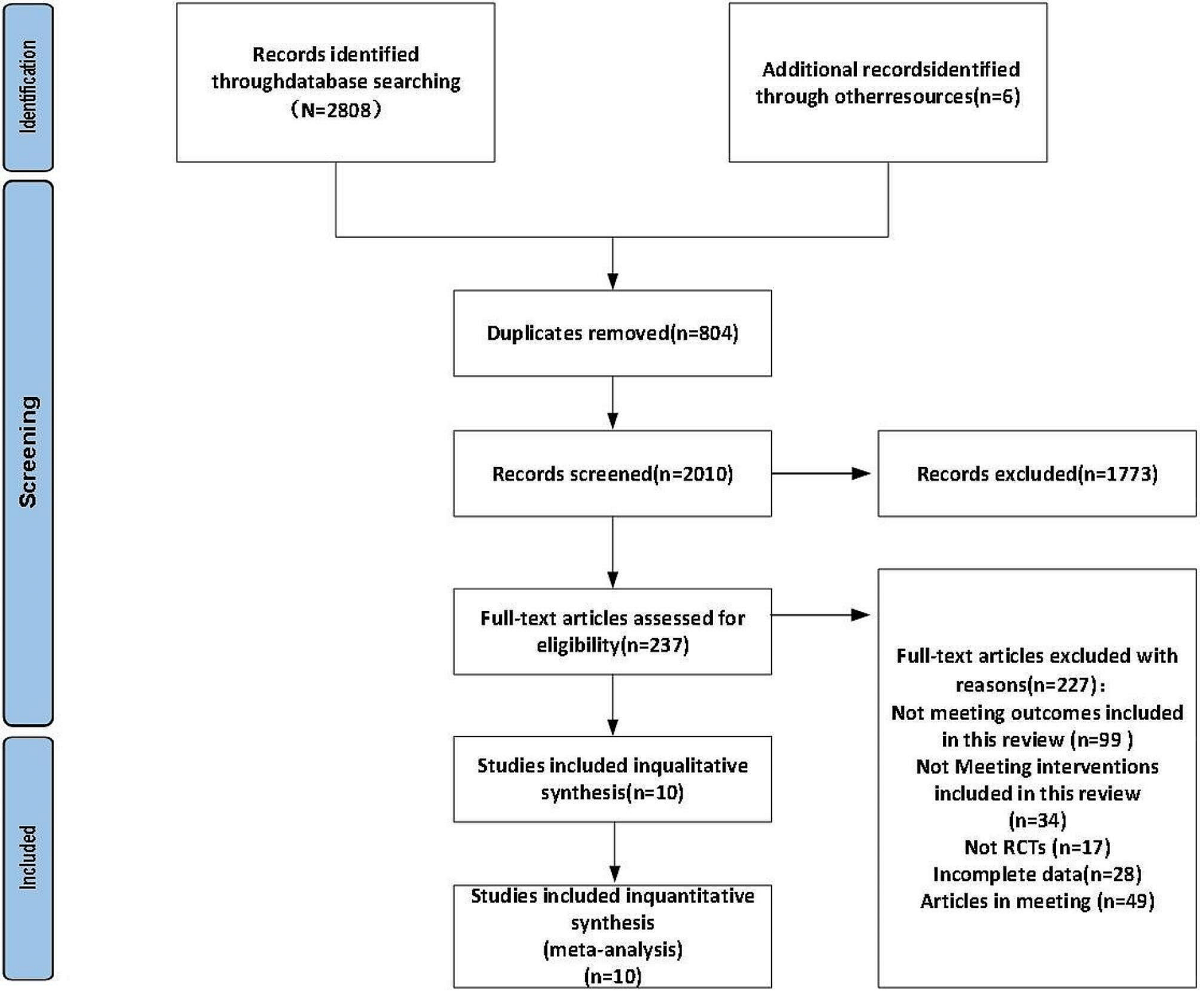
Flow diagram of the literature selection process

A total of 2808 articles were identified from four English databases: PubMed (*n* = 454), Embase (*n* = 521), Web of Science (*n* = 1038), and Cochrane (*n* = 795). After removing duplicates and a careful analysis of the titles and abstracts, A total of 237 articles were considered potential candidates. Additionally, After reading these full texts, six additional studies were added through references. Finally, 10 suitable articles [4, 30–38] were included in the study after a thorough full-text review (Fig. 1).

### Characteristics of the studies

A total of 10 randomized controlled trials including 859 participants (428 in the intervention group and 431 in the control group) were included. Five studies were conducted in China, two in South Korea, and one each in Sweden, Norway, and Italy. The participants ranged in age from 53 to 70.3 years. The intervention duration ranged from 5 weeks to 12 months, and the exercise frequency ranged from 2 to 7 days per week. Ten studies investigated aerobic exercise dosages, two focused on resistance exercise dosages, and four examined flexibility exercise dosages (Table 2). TG, TC, and LDL measurements were reported in seven studies involving 685 participants. Five studies provided HDL data for 391 participants, and BMI data were reported in seven studies encompassing 681 participants.

**Table 2:** Characteristics of the studies included in the meta-analysis

Author	Country	Year	Population	Age(mean + SD)	Total/male/female	Intervention	Control	Outcome
chen [30]	China	2020	AMI	T:59.98(10.86) C:61.49(11.54)	T:43/29/14 C:39/30/9	Baduanjin, Length of Intervention: 10 months Freq: 2 times a day Duration: 100–120 min	Standard care	BMI
Liu X [31]	China	2022	CHD	T:64.11(8.08) C:59.98(7.69)	T:46/34/12 C:49/37/12	Walking training, Length of Intervention: 12 weeks Freq: unclear Duration: unclear	Usual-care treatment	BMI、Lipid profiles(TG、LDL、HDL、TC)
Xiao [32]	China	2021	AMI	T:60.2 (9.2) C:58.7(8.8)	T:82/61/21 C:82/64/18	Walking training OR Cycling training, Length of Intervention: 12months Freq:3–5 times a week Duration:50–60 min	Usual-care treatment	BMI, Lipid profiles(TG、LDL、TC)
Lee [33]	Korea	2012	AMI	T: NA C: NA	T:22/NA/NA C:24/NA/NA	Walking training plus Flexibility training, Length of Intervention: 12weeks Freq:5 times a week Duration:50 min	Usual-care treatment	Lipid profiles(TG、LDL、HDL、TC)
Lee HY [34]	Korea	2013	AMI	T:58.8 (10.8) C:60.3(8.7)	T:37/30/7 C:39/24/15	supervised exercise Under prescription plus community-based and self-managed exercise, Length of Intervention:42 weeks Freq:3times a week Duration:50 min	Usual-care treatment	Lipid profiles(TG、LDL、HDL、TC)
Astengo [35]	Sweden	2010	stable angina	T:62 (7) C:65 (8)	T:28/20/8 C:28/24/4	Cycling training plus resistance training , Length of Intervention: 6months Freq:>2 times a week Duration:≥30 min	Usual-care treatment	Lipid profiles(TG、LDL、HDL、TC)
Belardinelli [36]	Italy	2001	CHD	T:53(11) C:59(10)	T:59/49/10 C:59/50/9	Cycling training plus Flexible training, Length of Intervention: 6months Freq:3 times a week Duration:50 min	Usual-care treatment	BMI, Lipid profiles(TG、LDL、HDL、TC)
Munk [4]	Norway	2009	CHD	T:57 (14) C:61 (10)	T:20/17/3 C:20/16/4	High-intensity interval training plus Flexible training、resistance training Length of Intervention: 6months Freq:3 times a week Duration:60 min	Usual-care treatment	BMI
Zhang [37]	China	2018	AMI	T:70.3 (10.7) C:69.8(10.4)	T:65/59/6 C:65/54/11	community-based training, Length of Intervention: 6months Freq:2 times a week Duration:15–50 min	Usual-care and conventional	BMI, Lipid profiles(TG、LDL、TC)
Xu [38]	China	2016	AMI	T:55.8 (9.7) C:55.6(8.9)	T:26/22/4 C:26/22/4	Walking training plus jogging、Flexible training , Length of Intervention: 5 weeks Freq: unclear Duration:30 min	Usual-care treatment	BMI

Note Unless stated otherwise, the figures represent averages (SDs). NR, not reported; T, experimental group; C, control group; BP, blood pressure

### Risk of bias assessment

**Fig. 2:**
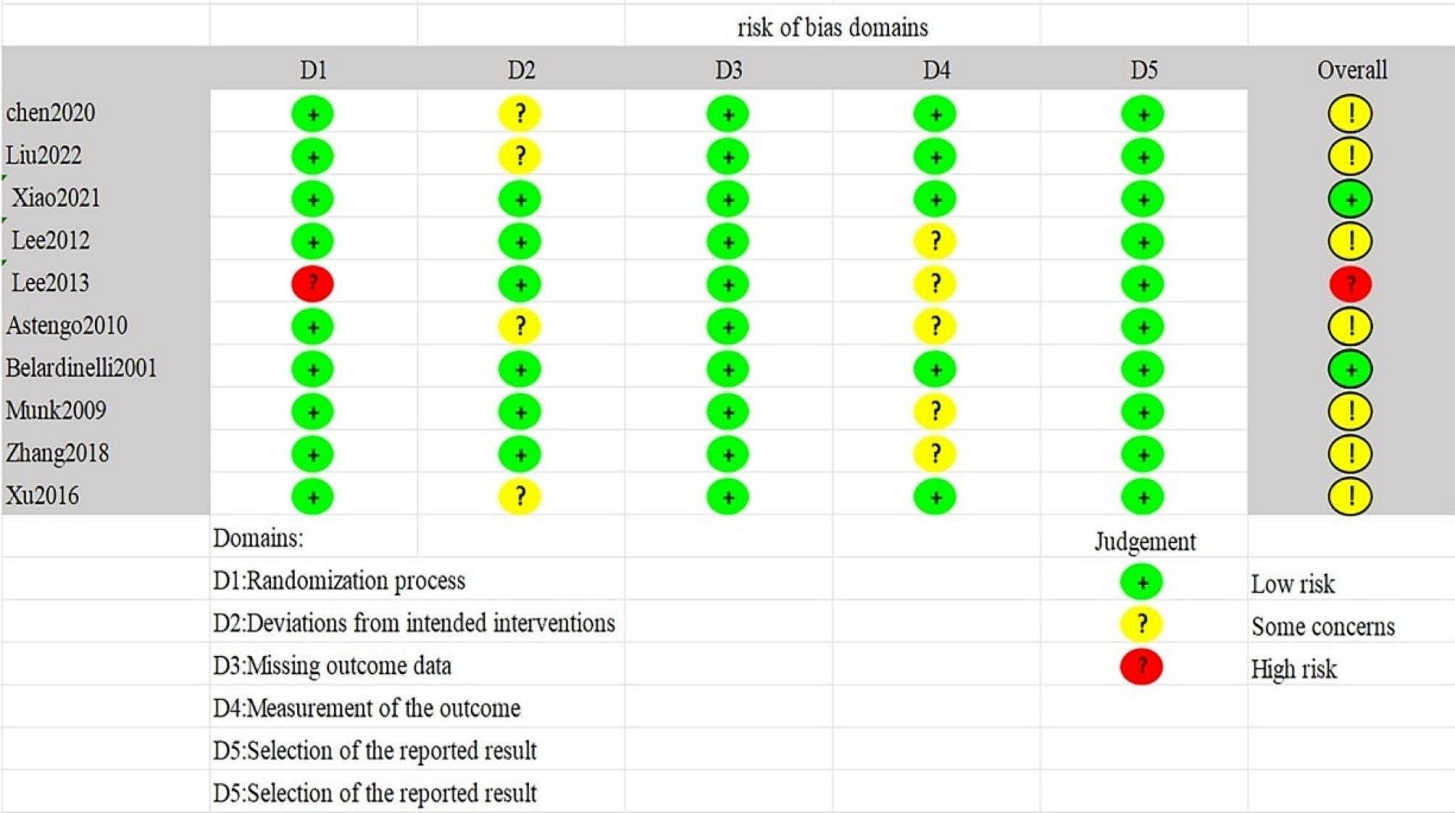
Graph showing the risk of bias of the included studies

**Fig. 3:**
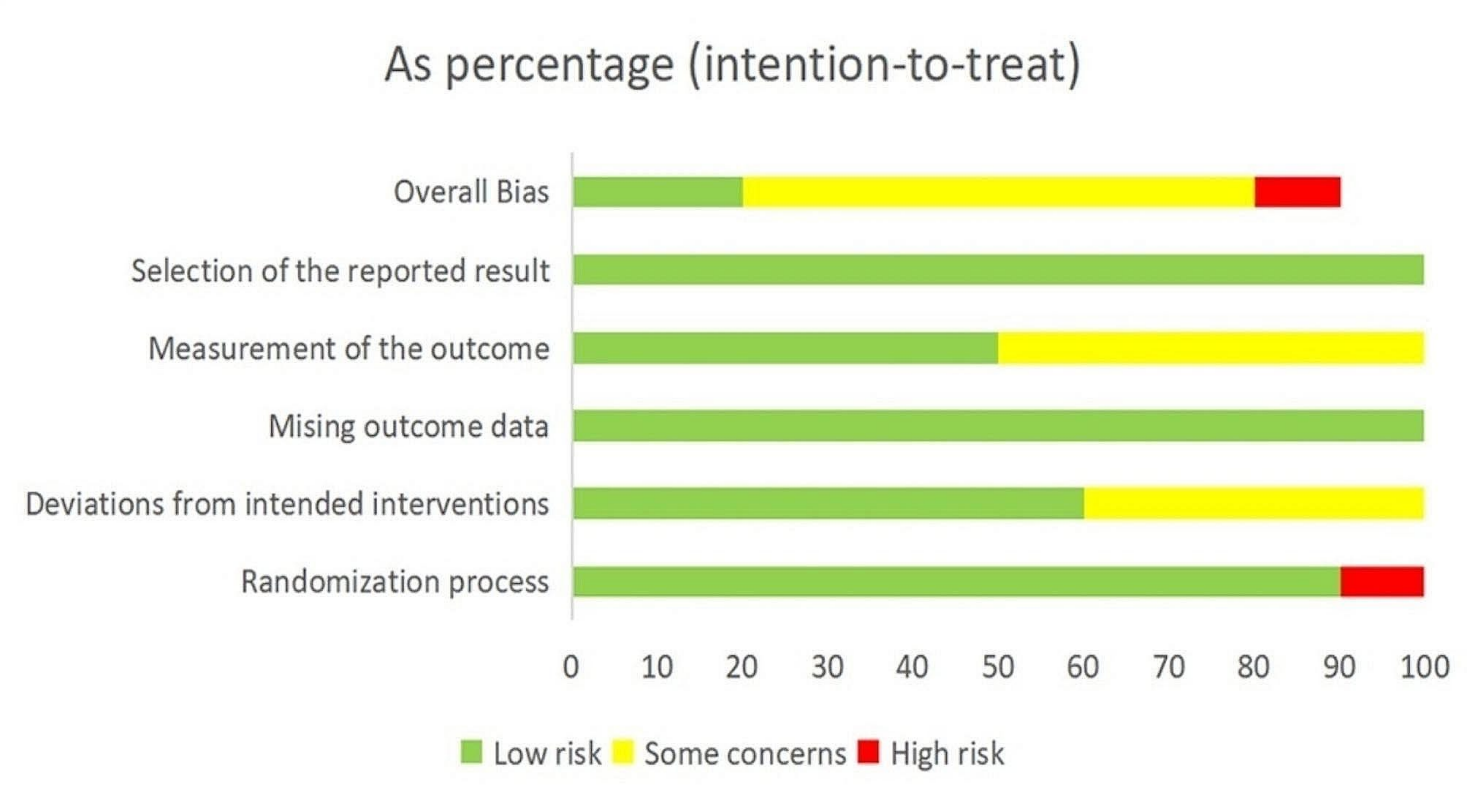
Graph showing the risk of bias assessment for the included RCTs

Five studies were classified as having a low risk of bias for the expected intervention measures. However, five studies deviated from the intended intervention groups due to participants’ awareness of their allocation. The number of people who participated in the study after the intervention was largely symmetrical or consistent with that at baseline, and only three studies employed blind assessors for outcome evaluations. Overall, two studies exhibited a low risk of bias, one study carried a high risk of bias, and seven studies were categorized as having an uncertain risk of bias (Figs. 2 and 3).

### Compliance with the acsm recommendations

Seven studies demonstrated a compliance rate of ≥ 70% with the ACSM recommendations, while three studies exhibited a compliance rate of < 70% (Table 3). This lower compliance was primarily attributed to mismatches between the exercise dosages and the ACSM recommendations, as well as a lack of necessary assessment information. The ACSM compliance rates across various outcome measures were as follows: Among studies using TG as an indicator, five exhibited high ACSM exercise compliance, while two showed lower or uncertain compliance. Similarly, for TC levels, five studies reported high ACSM compliance, and two studies reported lower or uncertain compliance. For LDL levels, five studies reported high ACSM compliance, and two studies reported lower or uncertain compliance. Regarding HDL levels as an indicator, four studies reported high ACSM compliance, and one study reported lower or uncertain compliance. For studies using BMI as a measure, four studies had higher ACSM compliance, and three studies exhibited lower or uncertain compliance.

**Table 3:**
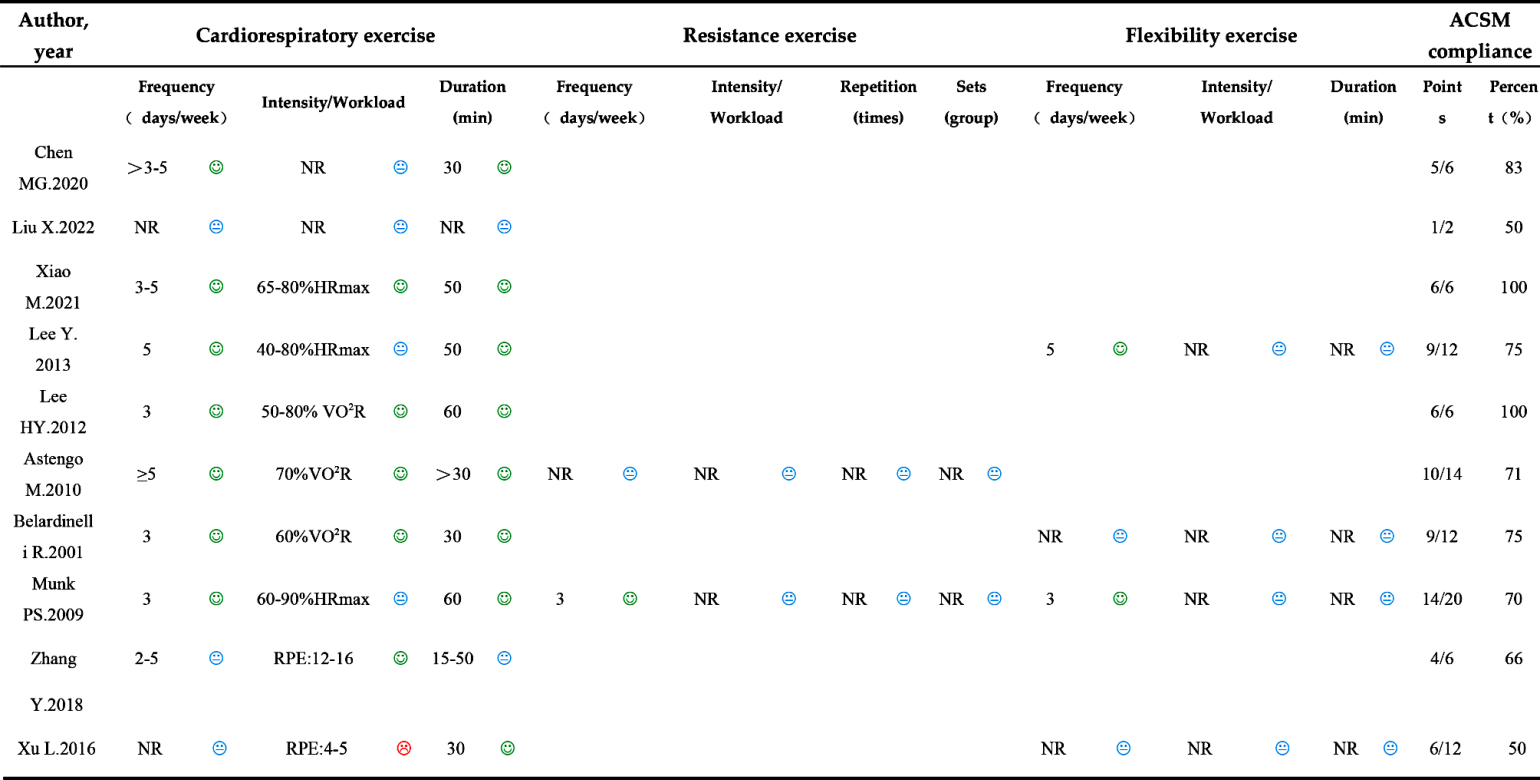
Assessment of ACSM compliance

## Meta-analysis

### Triglycerides

**Fig. 4:**
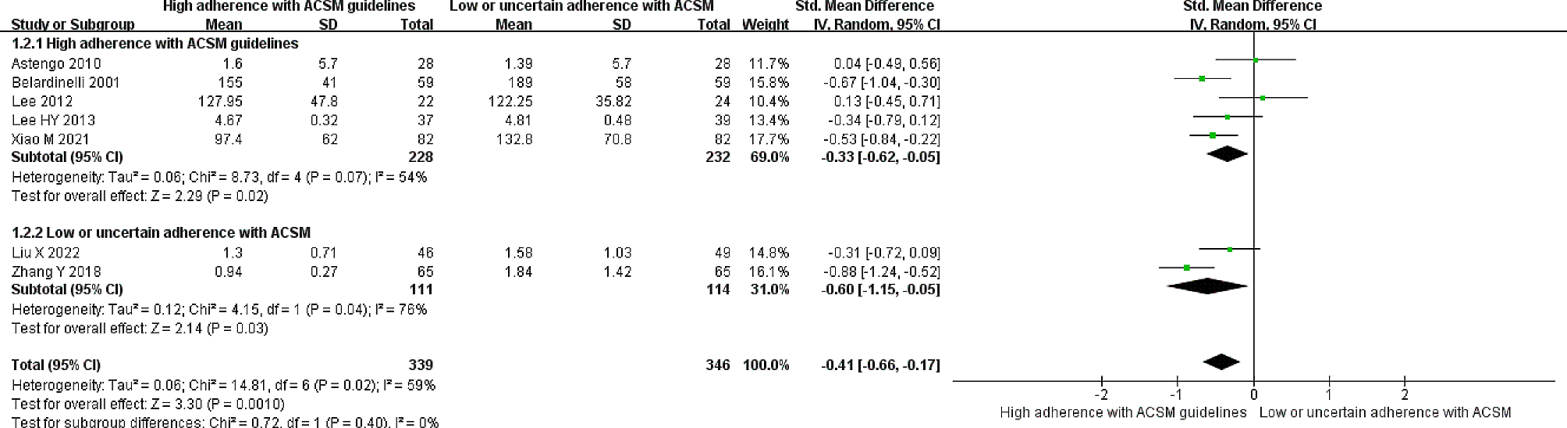
Forest plot of the effect of the exercise dosage on TG levels

Seven studies reported the correlation between TG levels and exercise. The heterogeneity test showed an *I*^*2*^ of 59% with a *P* value < 0.05, indicating the presence of heterogeneity; thus, a random-effects model was employed for the analysis. The meta-analysis revealed a significant reduction of 0.41 mmol/l in triglyceride levels in the intervention group compared to the nonexercise group (95% *CI*: -0.66, -0.17; *P* < 0.05). Studies were grouped based on their compliance with the ACSM-recommended exercise dosages to further explore the effect. Among these studies, five studies displayed high compliance with ACSM recommendations, and the results showed a significant reduction of 0.33 mmol/l in triglyceride levels (95% *CI*: -0.62, -0.05; *P* = 0.02). The other two studies exhibited low or uncertain compliance with ACSM recommendations, and the intervention group had a significant reduction of 0.60 mmol/l in triglyceride levels compared to the control group (95% *CI*: -1.15, -0.05; *P* = 0.03) (Fig. 4). Begg’s test (*P* = 0.024) and Egger’s test (*P* = 0.030) confirmed the presence of publication bias. The sensitivity analysis confirmed the robustness of the study results (Fig. 9).

### Total cholesterol

**Fig. 5:**
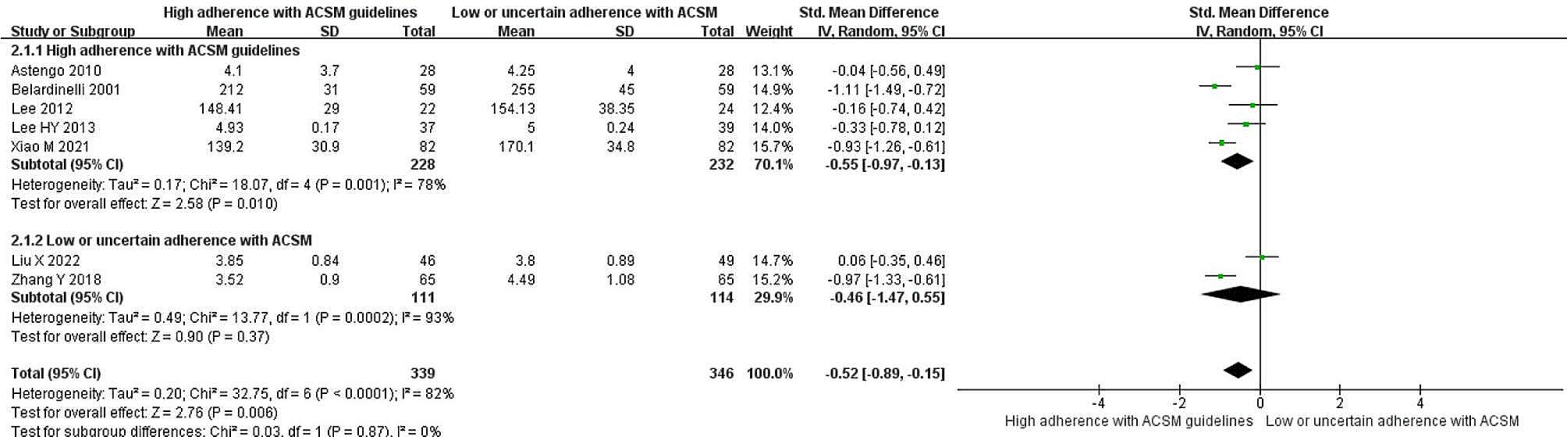
Forest plot of the effect of the exercise dosage on TC levels

Seven studies reported the correlation between TC levels and exercise. Heterogeneity testing yielded an *I*^*2*^ of 82% with a *P* value < 0.001, indicating significant heterogeneity; thus, a random effects model was used for the analysis. The meta-analysis revealed a significant reduction of 0.52 mmol/l in total cholesterol levels in the intervention group compared with the nonexercise group (95% *CI*: -0.89, -0.15; *P* < 0.05). Grouping the studies based on compliance with ACSM-recommended exercise dosages revealed that among the five studies with high compliance, a significant reduction of 0.55 mmol/l in total cholesterol levels (95% *CI*: -0.97, -0.13; *P* = 0.01) was observed. The two studies with low or uncertain compliance did not show a significant reduction in total cholesterol levels (95% *CI*: -1.47, 0.55; *P* = 0.37) (Fig. 5). Additionally, Begg’s test (*P* = 0.176) and Egger’s test (*P* = 0.094) did not confirm significant publication bias. The sensitivity analysis confirmed the robustness of the study results (Fig. 9).

### Low-density lipoprotein

**Fig. 6:**
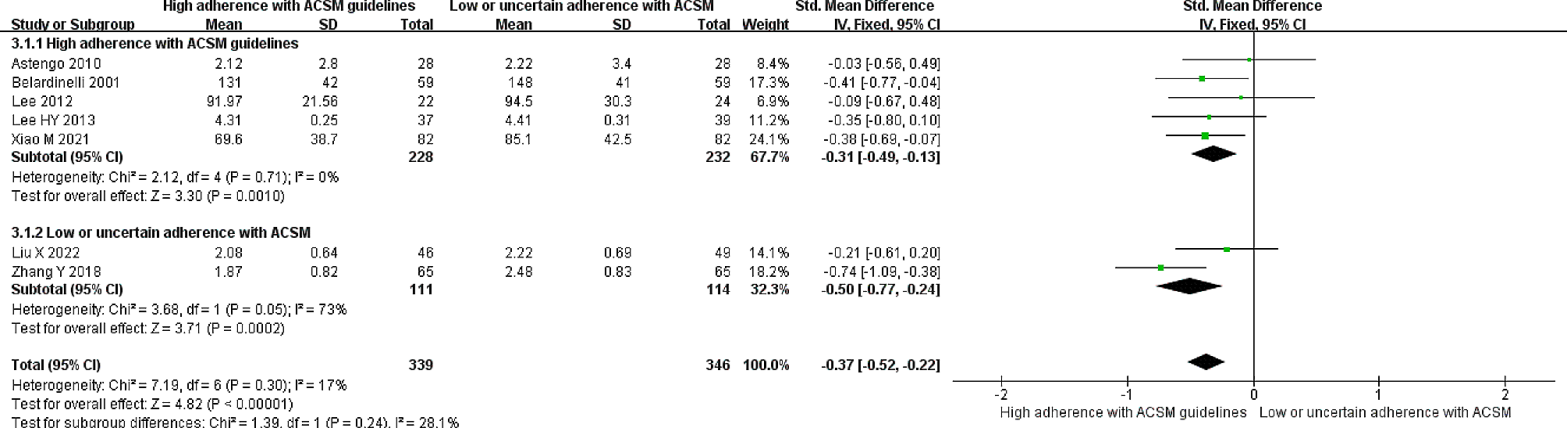
Forest plot of the effect of the exercise dosage on LDL levels

Seven studies reported the correlation between LDL levels and exercise. Evaluating the same treatment effects in the included studies and heterogeneity testing showed an *I*^*2*^ of 17% with a *P* value > 0.005, indicating low heterogeneity; thus, a fixed-effects model was employed for the analysis. The meta-analysis revealed a significant reduction of 0.37 mmol/l in LDL levels in the intervention group compared to the nonexercise group (95% *CI*: -0.52, -0.22; *P* < 0.05). When grouped based on compliance with ACSM-recommended exercise dosages, the five studies with high compliance showed a significant reduction of 0.31 mmol/l in LDL levels (95% *CI*: -0.49, -0.13; *P* = 0.001). The two studies with low or uncertain compliance exhibited a significant reduction of 0.50 mmol/l in LDL levels (95% *CI*: -0.77, -0.24; *P* = 0.0002) (Fig. 6). Additionally, Begg’s test (*P* = 0.051) and Egger’s test (*P* = 0.127) did not confirm significant publication bias. The sensitivity analysis confirmed the robustness of the study results (Fig. 9C).

### High-density lipoprotein

**Fig. 7:**
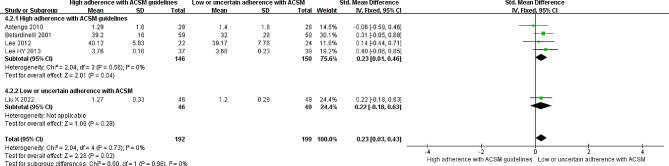
Forest plot of the effect of the exercise dosage on HDL levels

Five research reports explored the correlation between HDL levels and exercise. Heterogeneity testing revealed an *I*^*2*^ value of 0.0% with a *P* value greater than 0.005, indicating homogeneity across studies. Therefore, a fixed-effects model was employed for the analysis. The meta-analysis indicated a significant increase of 0.23 mmol/l in HDL levels in the intervention group compared to the nonexercise group (95% *CI*: 0.03, 0.43; *P* < 0.05). Further grouping based on compliance with the ACSM-recommended exercise intervention dosages was conducted. Among these reports, four studies reported high compliance with ACSM recommendations, with a significant increase of 0.23 mmol/l in HDL levels (95% *CI*: 0.01, 0.46; *P* = 0.04). Conversely, one study reported low or uncertain compliance with ACSM recommendations, revealing a nonsignificant increase of 0.22 mmol/l in HDL levels (95% *CI*: -0.18, 0.63; *P* = 0.28) (Fig. 7). Additionally, both Begg’s test (*P* = 0.327) and Egger’s test (*P* = 0.277) confirmed the absence of significant publication bias. The sensitivity analysis further corroborated the robustness of the study findings (Fig. 9).

### Body mass index

**Fig. 8:**
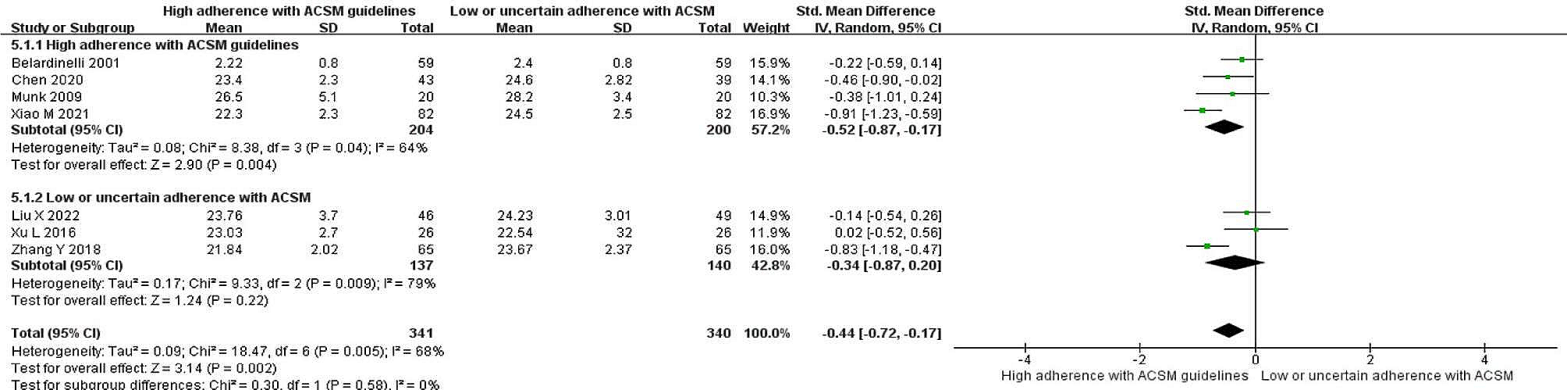
Forest plot of the effect of the exercise dosage on BMI

**Fig. 9:**
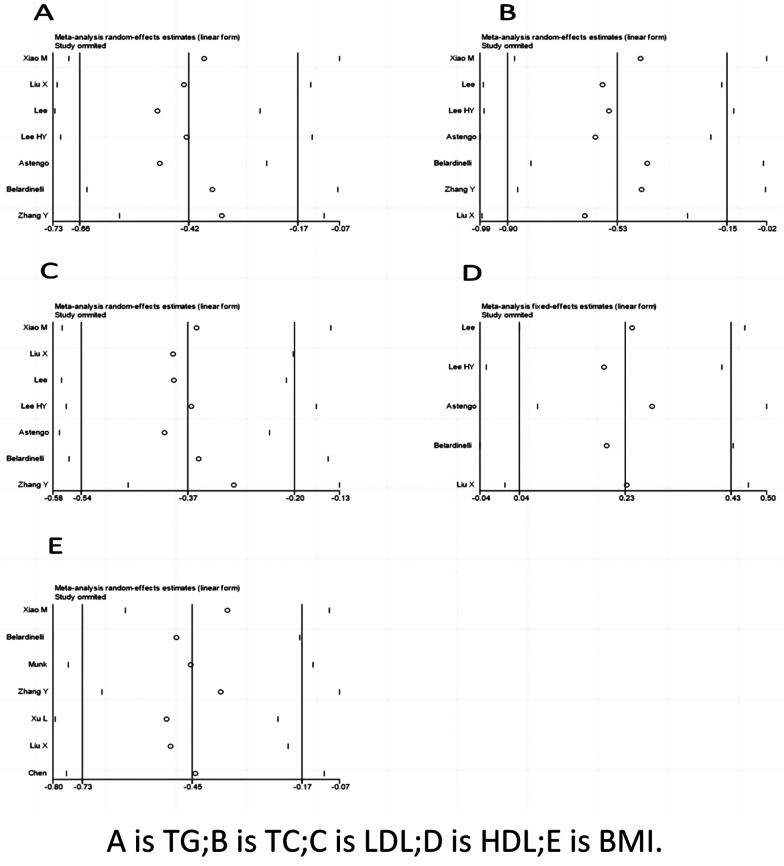
Sensitivity analysis of the meta-analysis of the effect of the exercise dosage on TG levels, TC levels, LDL levels, HDL levels, and BMI

Seven research reports examined the correlation between BMI and exercise. Heterogeneity testing yielded an *I*^*2*^ value of 68% with a *P* value less than 0.05, indicating heterogeneity across studies. Therefore, a random-effects model was employed for the analysis. The meta-analysis revealed a significant decrease of 0.44 mmol/l in BMI in the intervention group compared to the nonexercise group (95% *CI*: -0.72, -0.17; *P* < 0.05). Grouping based on compliance with the ACSM-recommended exercise intervention dosages was also conducted. Four of these studies reported high compliance with ACSM recommendations, showing a significant decrease of 0.52 mmol/l in BMI (95% *CI*: -0.87, -0.17; *P* = 0.004). Three studies reported low or uncertain compliance with ACSM recommendations, showing a nonsignificant decrease of 0.34 mmol/l in BMI (95% *CI*: -0.87, 0.20) (Fig. 8). Additionally, both Begg’s test (*P* = 0.099) and Egger’s test (*P* = 0.189) confirmed the absence of significant publication bias. The sensitivity analysis further corroborated the robustness of the study findings (Fig. 9E).

## Discussion

Studies have indicated that abnormal lipid metabolism is a primary factor influencing restenosis in revascularized coronary arteries [39]. Among these lipids, LDL is a crucial protective factor for atherosclerosis and cardiovascular diseases. For every 1 mmol/L reduction in the level of this indicator, a 20% reduction in the need for revascularization surgery and a 10% reduction in mortality have been reported [40]. HDL is a primary factor inducing atherosclerosis and is often accompanied by increased TG and LDL levels [41]. The TG/HDL ratio also plays a crucial role in the occurrence of ISR post-PCI [42]. Both the American Heart Association and the American College of Cardiology have recognized that am increased BMI strongly correlates with increased incidences of MACE and heart failure. Obesity has been identified as a modifiable risk factor for cardiovascular disease [43]. Typically, CHD patients with a higher BMI often have enlarged ventricular diameters, reduced diastolic function, more severe inflammatory responses, and a greater risk of developing MACE [44].Fatty acids stored in adipose tissue are released as triglycerides during lipolysis to promote fat oxidation in energy-consuming tissues. Fatty acid oxidation decreases after carbohydrate intake but increases during physical activity [45]. Disequilibrium between the production and utilization of fatty acids can have adverse effects on cardiovascular health and metabolism [46]. Acute increases in fatty acid levels in the human body are associated with impaired endothelium-mediated vasodilation, while chronic increases can contribute to dyslipidemia and lipid-induced toxicity in the heart [47]. Conversely, interventions that reduce fatty acid levels have shown potential benefits in improving metabolic health. Studies have confirmed that aerobic exercise can activate lipoprotein lipase activity, enabling muscles to fully absorb and utilize fatty acids and cholesterol and accelerating the transfer of phospholipids and cholesterol to HDL, thereby reducing total cholesterol, triglyceride, and LDL levels, and BMI [48]. This result suggests that exercise training is effective at improving blood lipid and BMI levels. However, exercise dosage is a topic worth exploring for the development of exercise prescriptions. For post-PCI patients, what dose of exercise will improve lipid metabolism and BMI remains unknown. This study synthesized different exercise patterns, intensity levels, exercise durations, and other relevant metrics from previous studies. The aim of this study was to clarify the effect of ACSM-recommended exercise doses on lipid metabolism and BMI in post-PCI patients and to confirm the practical application of ACSM-recommended exercise doses.

### Impact of high adherence to the acsm-recommended exercise dosage on tg levels

TGs are lipid molecules formed by the combination of three long-chain fatty acids and glycerol and account for the highest content of lipids in the human body. Their primary functions include supplying and storing energy, protecting internal organs, maintaining body temperature, and shaping the body [49].

The studies included in this review support the beneficial effects of exercise on TG levels in patients post-PCI. The subgroup analysis revealed that the improvement in triglyceride levels in patients with a high degree of adherence to the ACSM-recommended exercise dosage after PCI was not greater than that in patients with lower or uncertain ACSM compliance (SMD: -0.33 vs. -0.60). An analysis of the literature characteristics indicated that combining aerobic exercise, resistance training, and flexibility training had a weaker impact on lipid metabolism than a single exercise. The inconsistency with the report by RIMMER et al. [50] may be related to differences in the ages of the study participants and the unclear exercise training protocols in some studies. However, some studies have suggested that both moderate-intensity and low-intensity exercise can significantly reduce TG levels in patients with CHD, with cholesterol and blood sugar levels decreasing more rapidly in the moderate-intensity group [51]. In this review, the decrease in TG levels may not be consistent with this conclusion due to limitations in the clarity of the total exercise volume.

### Impact of high adherence to the acsm-recommended exercise dosage on tc levels

TC refers to the sum of cholesterol contained in various lipoproteins in the blood. TC is closely related to cardiovascular diseases such as CHD, atherosclerosis, and myocardial infarction [52]. Notably, a U-shaped correlation between total cholesterol levels and cardiovascular disease mortality was reported [53].

This study indicated that exercise improved total cholesterol levels in patients after PCI surgery, which is inconsistent with the findings of IGARASHI et al. [54]. The authors believe that this may be related to differences in research subjects and unclear exercise training protocols. Through a subgroup analysis, a high adherence to the exercise Dosage recommended by the ACSM had a better effect on improving total cholesterol levels in patients after PCI surgery than a lower or uncertain adherence to the ACSM recommendations (SMD: -0.55 vs. -0.46). This result may be because a high adherence to the exercise Dosage recommended by the ACSM can continuously activate lipoprotein lipase activity, enabling muscles to fully absorb and utilize cholesterol and promote the transfer of cholesterol to high-density lipoproteins. Alternatively, this result may be related to the stimulation of lecithin-cholesterol acyltransferase activity, leading to increased levels of esterified cholesterol.

### Impact of high adherence to the acsm-recommended exercise dosage on ldl levels

LDL is a lipoprotein particle responsible for transporting cholesterol to peripheral tissue cells. When LDL binds excessively to cholesterol, it forms LDL-C and accumulates on the arterial wall, eroding vascular endothelial cells and ultimately leading to atherosclerosis and thrombus formation [22].

This study demonstrated that exercise improved LDL levels in patients after PCI surgery. However, some studies have shown that exercise increases LDL levels [55], which is inconsistent with the results of this study. The subgroup analysis revealed that a high adherence to the exercise Dosage recommended by the ACSM had some positive effects on LDL levels in patients after PCI surgery. However, in this study, a high adherence to the ACSM-recommended exercise Dosage was not superior to a lower or uncertain adherence to the ACSM recommendations.

### Impact of high adherence to the acsm-recommended exercise dosage on hdl levels

HDL reduces blood coagulation by stimulating endothelial cells to secrete nitric oxide (NO) and suppresses inflammatory responses by inhibiting the expression of inflammatory factors in endothelial cells. Additionally, HDL clears excess cholesterol from macrophages in the arterial wall, thus reducing the risk of atherosclerosis, and is often considered an indicator of good health [56]. Recent studies have also documented that the implementation of exercise programs can effectively reduce the risk of atherosclerosis by enhancing the anti-inflammatory function of HDL [57].

This research demonstrated that exercise had a significant beneficial effect on HDL levels in patients after PCI, which is inconsistent with the findings of Milanovic [48]. Furthermore, a heterogeneity of 0% indicates a high degree of consistency among these studies. According to the subgroup analysis, a high adherence to the exercise Dosage recommended by the ACSM had some positive effects on HDL levels in patients after PCI surgery. This result may be related to the fact that a high adherence to the ACSM-recommended exercise Dosage can more effectively stimulate the body to produce positive physiological responses, including promoting cholesterol transport, improving vascular function, regulating lipid metabolism, reducing chronic inflammation, and affecting the endocrine system. On the other hand, the combined SMD for the lower or uncertain adherence to the ACSM-recommended exercise Dosage was 0.22 (95% *CI* -0.18, 0.63), with a confidence interval including zero, indicating uncertainty in the impact of this exercise Dosage on the target outcome.

### Impact of high adherence to the acsm-recommended exercise dosage on Bmi

Obesity is a common comorbidity in patients diagnosed with coronary artery disease [58]. BMI is a commonly used indicator to assess the degree of obesity. Studies have noted a linear relationship between BMI categories and the repetition of revascularization procedures in patients receiving PCI [59]. The risk of repeated revascularization procedures is lowest in underweight or normal-weight patients and highest in severely obese patients.

This study demonstrated that exercise had a significant beneficial effect on BMI. This finding is consistent with previous results [60]. According to the subgroup analysis, a high adherence to the exercise Dosage recommended by the ACSM had a greater effect on improving BMI in patients with CHD after PCI surgery than a lower or uncertain ACSM adherence (SMD: -0.52 vs. -0.34). The reason for this difference may be that a high adherence to the ACSM-recommended exercise Dosage can utilize metabolic effects for weight loss, resulting in greater energy expenditure potential and reduced visceral fat, leading to beneficial outcomes.

### Research advantages and limitations

This study is the first to investigate the relationship between the exercise Dosage and lipid metabolism in patients following PCI through a meta-analysis approach. Furthermore, based on the exercise Dosage recommended by the ACSM, the subgroup analysis revealed that strictly adhering to the ACSM’s recommendations has significant impacts on improving TC levels, HDL levels, and BMI. While this study provides valuable findings and contributions, certain limitations must be acknowledged. First, disparities exist in the research designs of the included studies, such as differences in the exercise intensity, frequency, intervention duration, and the combination of two training methods in combined training. These disparities may contribute to the heterogeneity in the results of this study. Second, most of the studies included did not include a clear description of the subjects’ diets during the exercise intervention, which could affect the outcome measures. Additionally, Some of the indicators included fewer studies with low or uncertain ACSM adherence, however, they did not affect the overall results.

## Conclusions

This review supports the hypothesis that exercise is an effective means to improve lipid metabolism and BMI in patients after PCI. In clinical work, the proper Dosage of exercise can significantly improve health benefits, whereas excessive or insufficient exercise may not achieve the desired health outcomes and may even have negative effects. In exploring the optimal Dosage of exercise for patients after PCI, this study revealed that strictly adhering to the exercise Dosage recommended by the ACSM has positive impacts on improving TC levels, TG levels, LDL levels, HDL levels, and BMI. Compared to exercise dosages with lower or uncertain ACSM compliance, strictly adhering to the ACSM recommendations significantly improved TC levels, HDL levels, and BMI. These results support the use of exercise dosages that strictly adhere to the ACSM recommendations as a treatment option for improving lipid metabolism in patients after PCI, maximizing the health benefits derived from exercise. However, compared to exercise dosages with lower or uncertain ACSM compliance, strictly adhering to the ACSM recommendations did not significantly improve TG or LDL levels. Therefore, future research will need more rigorous experimental designs and larger samples to confirm these findings.

### Electronic supplementary material

Below is the link to the electronic supplementary material.

Supplementary Material 1

## Data Availability

No datasets were generated or analysed during the current study.

## Abbreviations

PCI: Percutaneous Coronary Intervention
CHD: Coronary Heart Disease
ACSM: American College of Sports Medicine
HIIT: High-Intensity Interval training
TC: Total Cholesterol
TG: Triglycerides
LDL: Low Density lipoprotein
HDL: High Density lipoprotein
BMI: Body Mass Index

## References

[1] Writing Group M, Mozaffarian D, Benjamin EJ, Go AS, Arnett DK, Blaha MJ, et al. Executive Summary: Heart Disease and Stroke Statistics–2016 update: a Report from the American Heart Association. Circulation. 2016. 10.1161/CIR.0000000000000366. 10.1161/CIR.0000000000000366 26811276

[2] Doenst T, Thiele H, Haasenritter J, Wahlers T, Massberg S, Haverich A. The treatment of coronary artery disease. Dtsch Arztebl Int. 2022. 10.3238/arztebl.m2022.0277. 35912444 10.3238/arztebl.m2022.0277 PMC9835700

[3] Giustino G, Colombo A, Camaj A, Yasumura K, Mehran R, Stone GW, et al. Coronary In-Stent restenosis: JACC state-of-the-art review. J Am Coll Cardiol. 2022. 10.1016/j.jacc.2022.05.017. 35863852 10.1016/j.jacc.2022.05.017

[4] Munk PS, Staal EM, Butt N, Isaksen K, Larsen AI. High-intensity interval training may reduce in-stent restenosis following percutaneous coronary intervention with stent implantation A randomized controlled trial evaluating the relationship to endothelial function and inflammation. Am Heart J. 2009. 10.1016/j.ahj.2009.08.021. 19853690 10.1016/j.ahj.2009.08.021

[5] Chen HJ, Mo N, Zhang YF, Su GZ, Wu HD, Pei F. Role of Gene Polymorphisms/Haplotypes and plasma level of TGF-beta1 in susceptibility to In-Stent restenosis following coronary implantation of Bare Metal Stent in Chinese Han patients. Int Heart J. 2018. 10.1536/ihj.17-190. 29332922 10.1536/ihj.17-190

[6] Ullrich H, Olschewski M, Munzel T, Gori T. Coronary In-Stent restenosis: predictors and treatment. Dtsch Arztebl Int. 2021. 10.3238/arztebl.m2021.0254. 34379053 10.3238/arztebl.m2021.0254 PMC8715314

[7] Cai A, Li L, Zhang Y, Mo Y, Li Z, Mai W, et al. Baseline LDL-C and lp(a) elevations portend a high risk of coronary revascularization in patients after stent placement. Dis Markers. 2013. 10.1155/2013/472845. 24367139 10.1155/2013/472845 PMC3866824

[8] Gai MT, Zhu B, Chen XC, Liu F, Xie X, Gao XM, et al. A prediction model based on platelet parameters, lipid levels, and angiographic characteristics to predict in-stent restenosis in coronary artery disease patients implanted with drug-eluting stents. Lipids Health Dis. 2021. 10.1186/s12944-021-01553-2. 34587955 10.1186/s12944-021-01553-2 PMC8480001

[9] Li Y, Jin P, Hou F, Zhou Y. Association between TG-to-HDL-C ratio and In-Stent stenosis under Optical Coherence Tomography Guidance. J Med Syst. 2018. 10.1007/s10916-018-1119-y. 30460580 10.1007/s10916-018-1119-y

[10] Kannisto K, Gafvels M, Jiang ZY, Slatis K, Hu X, Jorns C, et al. LXR driven induction of HDL-cholesterol is independent of intestinal cholesterol absorption and ABCA1 protein expression. Lipids. 2014. 10.1007/s11745-013-3853-8. 24163219 10.1007/s11745-013-3853-8

[11] Chen Z, Peto R, Collins R, MacMahon S, Lu J, Li W. Serum cholesterol concentration and coronary heart disease in population with low cholesterol concentrations. BMJ. 1991. 10.1136/bmj.303.6797.276. 1888927 10.1136/bmj.303.6797.276 PMC1670480

[12] Cohen JCBE, Mosley TH Jr, Hobbs HH, Boerwinkle E, Mosley TH Jr, Hobbs HH. N Engl J Med. 2006. 10.1056/NEJMoa054013. 17135593 10.1056/NEJMoa054013

[13] van de Woestijne AP, Wassink AM, Monajemi H, Liem AH, Nathoe HM, van der Graaf Y, et al. Plasma triglyceride levels increase the risk for recurrent vascular events independent of LDL-cholesterol or nonHDL-cholesterol. Int J Cardiol. 2013. 10.1016/j.ijcard.2012.01.008. 22265582 10.1016/j.ijcard.2012.01.008

[14] Zhang L, Yuan F, Liu P, Fei L, Huang Y, Xu L, et al. Association between PCSK9 and LDLR gene polymorphisms with coronary heart disease: case-control study and meta-analysis. Clin Biochem. 2013. 10.1016/j.clinbiochem.2013.01.013. 23380588 10.1016/j.clinbiochem.2013.01.013

[15] Niebauer J, Hambrecht R, Velich T, Hauer K, Marburger C, Kalberer B, et al. Attenuated progression of coronary artery disease after 6 years of multifactorial risk intervention: role of physical exercise. Circulation. 1997. 10.1161/01.cir.96.8.2534. 9355890 10.1161/01.cir.96.8.2534

[16] Young JL, Rhon DI, Cleland JA, Snodgrass SJ. The influence of Exercise Dosing on outcomes in patients with knee disorders: a systematic review. J Orthop Sports Phys Ther. 2018. 10.2519/jospt.2018.7637. 29320945 10.2519/jospt.2018.7637

[17] Wasfy MM, Baggish AL. Exercise Dose in Clinical Practice. Circulation. 2016. 10.1161/CIRCULATIONAHA.116.018093. 27267537 10.1161/CIRCULATIONAHA.116.018093 PMC4902280

[18] Gordon B, Chen S, Durstine JL. The effects of exercise training on the traditional lipid profile and beyond. Curr Sports Med Rep. 2014. 10.1249/JSR.0000000000000073. 25014391 10.1249/JSR.0000000000000073

[19] Rimmer JH, Rauworth AE, Wang EC, Nicola TL, Hill B. A preliminary study to examine the effects of aerobic and therapeutic (nonaerobic) exercise on cardiorespiratory fitness and coronary risk reduction in stroke survivors. Arch Phys Med Rehabil. 2009. 10.1016/j.apmr.2008.07.032. 19254604 10.1016/j.apmr.2008.07.032

[20] Uddin J, Zwisler AD, Lewinter C, Moniruzzaman M, Lund K, Tang LH, et al. Predictors of exercise capacity following exercise-based rehabilitation in patients with coronary heart disease and heart failure: a meta-regression analysis. Eur J Prev Cardiol. 2016. 10.1177/2047487315604311. 26330205 10.1177/2047487315604311

[21] van der Meer S, Zwerink M, van Brussel M, van der Valk P, Wajon E, van der Palen J. Effect of outpatient exercise training programmes in patients with chronic heart failure: a systematic review. Eur J Prev Cardiol. 2012. 10.1177/1741826711410516. 22988592 10.1177/1741826711410516

[22] Durstine JL, Grandjean PW, Davis PG, Ferguson MA, Alderson NL, DuBose KD. Blood lipid and lipoprotein adaptations to exercise: a quantitative analysis. Sports Med. 2001. 10.2165/00007256-200131150-00002. 11735685 10.2165/00007256-200131150-00002

[23] Garber CE, Blissmer B, Deschenes MR, Franklin BA, Lamonte MJ, Lee IM, et al. American College of Sports Medicine position stand. Quantity and quality of exercise for developing and maintaining cardiorespiratory, musculoskeletal, and neuromotor fitness in apparently healthy adults: guidance for prescribing exercise. Med Sci Sports Exerc. 2011. 10.1249/MSS.0b013e318213fefb. 21694556 10.1249/MSS.0b013e318213fefb

[24] Muros Molina JJ, Oliveras Lopez MJ, Mayor Reyes M, Reyes Burgos T. Lopez Garcia De La Serrana. Influence of physical activity and dietary habits on lipid profile, blood pressure and BMI in subjects with metabolic syndrome. Nutr Hosp. 2011. 10.1590/S0212-16112011000500027. 22072359 10.1590/S0212-16112011000500027

[25] Higgins JPTAD, Gotzsche PC et al. The Cochrane collaboration’s tool for assessing risk of bias in randomised trials. BMJ. 2011. 10.1136/bmj.d5928 PMC3196245 22008217

[26] Sterne JACSJ, Page MJ et al. RoB 2: a revised tool for assessing risk of bias in randomised trials. BMJ. 2019. 10.1136/bmj.l4898 31462531

[27] Cumpston M, Li T, Page MJ, Chandler J, Welch VA, Higgins JP, et al. Updated guidance for trusted systematic reviews: a new edition of the Cochrane Handbook for Systematic Reviews of Interventions. Cochrane Database Syst Rev. 2019. 10.1002/14651858.ED000142. 31643080 10.1002/14651858.ED000142 PMC10284251

[28] Deeks JJHJ, Altman DG,Chapter 9: Analysing data and undertaking meta-analyses. In:, Higgins JP, Green S, editors. Cochrane Handbook for Systematic Reviews of Interventions Version 5.1.0 (updated March 2011). The Cochrane Collaboration, 2011. doi:Available from www.cochrane-handbook.org.

[29] Nikolakopoulou A, Mavridis D, Salanti G. How to interpret meta-analysis models: fixed effect and random effects meta-analyses. Evid Based Ment Health. 2014. 10.1136/eb-2014-101794. 24778439 10.1136/eb-2014-101794 PMC13404559

[30] Chen MG, Liang X, Kong L, Wang J, Wang F, Hu X, et al. Effect of Baduanjin Sequential Therapy on the quality of life and cardiac function in patients with AMI after PCI: a Randomized Controlled Trial. Evid Based Complement Alternat Med. 2020. 10.1155/2020/8171549. 32714423 10.1155/2020/8171549 PMC7355341

[31] Liu X, Zhou W, Fan W, Li A, Pang J, Chen Z, et al. The benefit of exercise rehabilitation guided by 6-minute walk test on lipoprotein-associated phospholipase A2 in patients with coronary heart disease undergoing percutaneous coronary intervention: a prospective randomized controlled study. BMC Cardiovasc Disord. 2022. 10.1186/s12872-021-02430-7. 35430800 10.1186/s12872-021-02430-7 PMC9014591

[32] Xiao M, Li Y, Guan X. Community-based physical Rehabilitation after Percutaneous Coronary intervention for Acute myocardial infarction. Tex Heart Inst J. 2021. 10.14503/THIJ-19-7103. 34139763 10.14503/THIJ-19-7103 PMC8262830

[33] Lee HY, Kim JH, Kim BO, Byun YS, Cho S, Goh CW, et al. Regular exercise training reduces coronary restenosis after percutaneous coronary intervention in patients with acute myocardial infarction. Int J Cardiol. 2013. 10.1016/j.ijcard.2012.06.122. 22795710 10.1016/j.ijcard.2012.06.122

[34] LEE Y J.I. JUS. Impact of Home Exercise training on patients with Acute myocardial infarction. JOURNAL OF PHYSICAL THERAPY SCIENCE.; 2012.

[35] Astengo M, Dahl A, Karlsson T, Mattsson-Hulten L, Wiklund O, Wennerblom B. Physical training after percutaneous coronary intervention in patients with stable angina: effects on working capacity, metabolism, and markers of inflammation. Eur J Cardiovasc Prev Rehabil. 2010. 10.1097/HJR.0b013e3283336c8d. 20560169 10.1097/HJR.0b013e3283336c8d

[36] Belardinelli R, Paolini I, Cianci G, Piva R, Georgiou D, Purcaro A. Exercise training intervention after coronary angioplasty: the ETICA trial. J Am Coll Cardiol. 2001. 10.1016/s0735-1097(01)01236-0. 11401128 10.1016/s0735-1097(01)01236-0

[37] Zhang Y, Cao H, Jiang P, Tang H. Cardiac rehabilitation in acute myocardial infarction patients after percutaneous coronary intervention: a community-based study. Med (Baltim). 2018. 10.1097/MD.0000000000009785. 10.1097/MD.0000000000009785 PMC5841979 29465559

[38] Xu L, Cai Z, Xiong M, Li Y, Li G, Deng Y, et al. Efficacy of an early home-based cardiac rehabilitation program for patients after acute myocardial infarction: a three-dimensional speckle tracking echocardiography randomized trial. Med (Baltim). 2016. 10.1097/MD.0000000000005638. 10.1097/MD.0000000000005638 PMC5207550 28033254

[39] zhou GR. Effect of integrated Chinese and western medicine therapy on blood lipid and blood rhein patients with qi deficiency and blood stasis after PCI for coronary heart disease. Emergency Department of Traditional Chinese Medicine in China; 2015.

[40] Zhao XH. Efficacy of probucol and rosuvastatin in the treatment of acute cerebral infarction and its effect on lipid metabolism. Internal Medicine.; 2018.

[41] Puri R, Nissen SE, Shao M, Ballantyne CM, Barter PJ, Chapman MJ, et al. Antiatherosclerotic effects of long-term maximally intensive statin therapy after acute coronary syndrome: insights from study of Coronary Atheroma by Intravascular Ultrasound: Effect of Rosuvastatin Versus Atorvastatin. Arterioscler Thromb Vasc Biol. 2014. 10.1161/ATVBAHA.114.303932. 25212234 10.1161/ATVBAHA.114.303932

[42] Zhao HL, Shao WY. Jun,l L. Major nosocomial adverse vascular events after PCI in a patient with acute coronary syndrome the relationship between incidence and serum TG / HDLC. PLA J Prev Med. 2019.

[43] Writing Committee M, Virani SS, Newby LK, Arnold SV, Bittner V, Brewer LC, AHA/ACC/ACCP/, ASPC/NLA/PCNA Guideline for the Management of Patients With Chronic Coronary Disease. A Report of the American Heart Association/American College of Cardiology Joint Committee on Clinical Practice Guidelines. J Am Coll Cardiol. 2023. 10.1016/j.jacc.2023.04.003. 10.1016/j.jacc.2023.04.003 37480922

[44] Zhao DG. Prognosis difference analysis of patients undergoing coronary stent implantation with different body mass. Chin J Clin. 2018.

[45] Spriet LL. New insights into the Interaction of Carbohydrate and Fat Metabolism during Exercise. Sports Med 2014. 10.1007/s40279-014-0154-1. 10.1007/s40279-014-0154-1 PMC4008806 24791920

[46] Steinberg HO, Tarshoby M, Monestel R, Hook G, Cronin J, Johnson A, et al. Elevated circulating free fatty acid levels impair endothelium-dependent vasodilation. J Clin Invest. 1997. 10.1172/JCI119636. 9276741 10.1172/JCI119636 PMC508300

[47] Gl S. Ectopic Fat in insulin resistance, Dyslipidemia, and Cardiometabolic Disease. N Engl J Med. 2014. 10.1056/NEJMc1412427. 10.1056/NEJMc1412427

[48] Milanovic Z, Pantelic S, Covic N, Sporis G, Mohr M, Krustrup P. Broad-spectrum physical fitness benefits of recreational football: a systematic review and meta-analysis. Br J Sports Med. 2019. 10.1136/bjsports-2017-097885. 29371223 10.1136/bjsports-2017-097885 PMC6662951

[49] Welty FK. How do elevated triglycerides and low HDL-cholesterol affect inflammation and atherothrombosis? Curr Cardiol Rep. 2013. 10.1007/s11886-013-0400-4. 23881582 10.1007/s11886-013-0400-4 PMC4465984

[50] Fletcher GF, Ades PA, Kligfield P, Arena R, Balady GJ, Bittner VA, et al. Exercise standards for testing and training: a scientific statement from the American Heart Association. Circulation. 2013. 10.1161/CIR.0b013e31829b5b44. 23877260 10.1161/CIR.0b013e31829b5b44

[51] Wang Yue LJ, Zhang, Yong. Effect of exercise rehabilitation intensity on cardiac function and serum lipids in elderly. Anhui Med. 2018. doi:10.

[52] Li JX, Cao J, Lu XF, Chen SF, Yu DH, Duan XF, et al. The effect of total cholesterol on myocardial infarction in Chinese male hypertension population. Biomed Environ Sci. 2010. 10.1016/S0895-3988(10)60029-3. 20486434 10.1016/S0895-3988(10)60029-3

[53] Zhu Y, Lu J-M, Yu Z-B, Li D, Wu M-Y, Shen P, et al. Intra-individual variability of total cholesterol is associated with cardiovascular disease mortality: a cohort study. Nutr Metabolism Cardiovasc Dis. 2019. 10.1016/j.numecd.2019.07.007. 10.1016/j.numecd.2019.07.007 31383502

[54] Igarashi Y, Akazawa N, Maeda S. Effects of Aerobic Exercise alone on lipids in healthy East asians: a systematic review and Meta-analysis. J Atheroscler Thromb. 2019. 10.5551/jat.45864. 30381613 10.5551/jat.45864 PMC6514176

[55] Donovan O G., Owen A, Bird SR, Kearney EM, Nevill A.M., Jones D.W., et al. Changes in cardiorespiratory fitness and coronary heart disease risk factors following 24 wk of moderate- or high-intensity exercise of equal energy cost. J Appl Physiol (1985). 2005. 10.1152/japplphysiol.01310.2004. 10.1152/japplphysiol.01310.2004 15640382

[56] W. PW. High-density lipoprotein, low-density lipoprotein and coronary artery disease. Am J Cardiol. 1990. 10.1016/0002-9149(90)90562-f. 10.1016/0002-9149(90)90562-f 2203248

[57] Ahn NK. Kijin. High-density lipoprotein cholesterol (HDL-C) in cardiovascular disease: effect of exercise training. Integr Med Res. 2016. 10.1016/j.imr.2016.07.001. 28462120 10.1016/j.imr.2016.07.001 PMC5390423

[58] Ma WQSX, Wang Y, Han XQ, Zhu Y, Liu NF. Does body mass index truly affect mortality and cardiovascular outcomes in patients after coronary revascularization with percutaneous coronary intervention or coronary artery bypass graft? A systematic review and network meta-analysis. Obes Rev. 2018. 10.1111/obr.12713. 30035367 10.1111/obr.12713

[59] Wang ZJ, Gao F, Cheng WJ, Yang Q. Zhou. Body Mass Index and repeat revascularization after percutaneous coronary intervention: a Meta-analysis. Can J Cardiol. 2015. 10.1016/j.cjca.2015.01.031. 25921864 10.1016/j.cjca.2015.01.031

[60] Li J, Gao X, Hao X, Kantas D, Mohamed EA, Zheng X, et al. Yoga for secondary prevention of coronary heart disease: a systematic review and meta-analysis. Complement Ther Med. 2021. 10.1016/j.ctim.2020.102643. 33338581 10.1016/j.ctim.2020.102643

