# Supplementary material for: Impact of exercise dosages based on American College of Sports Medicine recommendations on lipid metabolism in patients after PCI: a systematic review and meta-analysis of randomized controlled trials
Source: Lipids Health Dis. 2024 Jul 24;23:226. doi: 10.1186/s12944-024-02210-0 (PMC11267757; doi:10.1186/s12944-024-02210-0)
Supplement: Supplementary file 1 — Supplementary Material 1 [file 12944_2024_2210_MOESM1_ESM.docx]

***Supplementary Material***

**Impact of Exercise Doses Based on ACSM Recommendations on Lipid Metabolism in Patients after PCI: A Systematic Review and Meta-Analysis of Randomized Controlled Trials**

**Qing Wen,MB^#a^. Xiao-Rong Mao, MD^#b^.Qun-Hua Ma,MB^a*^. Xiao-Li Tang, MB^c*^. Juan Wen, MB^d^. Xiao-Juan Yang, MB^e^. Juan Chen,MB^f^. Hu-Kui Han,MB^a^.**

***Corresponding Author: Qun-Hua Ma, 1203370164@qq.com;**

**Xiao-Li Tang, 1585470513@qq.com;**

**Appendix**  Search Strategy

| Database | Search strategy | amount |
| --- | --- | --- |
|  | PubMed |  |
| #1 | Search: "Percutaneous CoronaryIntervention"[Mesh] | 66014 |
| #2 | Search: ((((((((((((Percutaneous Coronary Intervention[Title/Abstract])) OR (Coronary Intervention, Percutaneous[Title/Abstract])) OR (Coronary Interventions, Percutaneous[Transliterated Title])) OR (Intervention, Percutaneous Coronary[Title/Abstract])) OR (Interventions, Percutaneous Coronary[Title/Abstract])) OR (Percutaneous Coronary Interventions[Title/Abstract])) OR (Percutaneous Coronary Revascularization[Title/Abstract])) OR (Coronary Revascularization, Percutaneous[Title/Abstract])) OR (Coronary Revascularizations, Percutaneous[Title/Abstract])) OR ( Percutaneous Coronary Revascularizations[Title/Abstract])) OR (Revascularization, Percutaneous Coronary[Title/Abstract])) OR (Revascularizations, Percutaneous Coronary[Title/Abstract]) | 47123 |
| #3 | #1 OR #2 | 82193 |
| #4 | Search: "Exercise"[Mesh] | 251684 |
| #5 | Search: (((((((((((((((Exercises[Title/Abstract]) OR (Physical Activity[Title/Abstract])) OR (physical exercise[Title/Abstract])) OR (Training[Title/Abstract])) OR (Trainings[Title/Abstract])) OR (Motor Activity[Title/Abstract])) OR (Tai Chi[Title/Abstract])) OR (Vibration[Title/Abstract])) OR (yoga[Title/Abstract])) OR (wuqinxi[Title/Abstract])) OR (baduanjin[Title/Abstract])) OR (yijinjing[Title/Abstract])) OR (kickboxing[Title/Abstract])) OR (Pilates[Title/Abstract])) OR (Balance[Title/Abstract])) OR (Resistance[Title/Abstract]) | 1977872 |
| #6 | #4 OR #5 | 2099909 |
| #7 | Search: ((((Randomized controlled trial[Publication Type]) OR (controlled clinical trial[Publication Type])) OR (randomized[Title/Abstract])) OR (placebo[Title/Abstract])) OR (randomly[Title/Abstract]) | 1365868 |
| #8 | #3 AND #6 AND #7 | 454 |
|  | Embase |  |
| #1 | 'Percutaneous Coronary Intervention'/exp OR 'Coronary Intervention, Percutaneous':ab,ti OR 'Coronary Interventions, Percutaneous':ab,ti OR 'Intervention, Percutaneous Coronary':ab,ti OR 'Interventions, Percutaneous Coronary':ab,ti OR 'Percutaneous Coronary Revascularization':ab,ti OR 'Percutaneous Coronary Revascularizations':ab,ti OR 'Coronary Revascularization, Percutaneous':ab,ti OR 'Percutaneous Coronary Revascularizations':ab,ti OR 'Revascularization, Percutaneous Coronary':ab,ti OR 'Revascularizations, Percutaneous Coronary':ab,ti | 130423 |
| #2 | 'exercis'/exp OR 'exercise':ab,ti OR 'exercisesn':ab,ti OR 'physical activity':ab,ti OR 'physical exercise':ab,ti OR 'raining':ab,ti OR 'trainingsl':ab,ti OR 'motor activity':ab,ti OR 'tai chi':ab,ti OR 'vibration':ab,ti OR 'yoga':ab,ti OR 'wuqinxi':ab,ti OR 'baduanjin':ab,ti OR 'yijinjing':ab,ti OR 'kickboxing':ab,ti OR 'pilates':ab,ti OR 'balance':ab,ti OR 'resistance':ab,ti | 2126288 |
| #3 | [controlled clinical trial]/lim OR [randomized controlled trial]/lim | 980808 |
| #4 | #1 AND #2 AND #3 | 521 |
|  | Web of Science |  |
| #1 | ALL=(percutaneous coronary intervention* OR Coronary Intervention, Percutaneous OR Intervention, Percutaneous Coronary OR LPercutaneous Coronary Revascularization OR Coronary Revascularization, Percutaneous OR Revascularization, Percutaneous Coronary) | 69184 |
| #2 | ((((((((((((((((ALL=(Exercise)) OR TS=(Exercises)) OR TS=(Physical Activity )) OR TS=(physical exercise)) OR TS=( Training )) OR TS=(Trainings)) OR TS=( Motor Activity)) OR TS=( Tai Chi )) OR TS=(Vibration )) OR TS=(yoga )) OR TS=( wuqinxi )) OR TS=( baduanjin )) OR TS=(yijinjing )) OR TS=(kickboxing )) OR TS=(Pilates)) OR TS=( Balance)) OR TS=( Resistance) | 5571460 |
| #3 | ((((TS=(Randomized controlled trial )) OR TS=(controlled clinical trial )) OR TS=( randomized )) OR TS=(placebo )) OR TS=( randomly) | 1584625 |
| #5 | #1 AND #2 AND #3 | 1038 |
|  | Cochrane |  |
| #1 | (percutaneous coronary intervention):ti,ab,kw OR (Coronary Intervention, Percutaneous):ti,ab,kw OR (Coronary Interventions, Percutaneous):ti,ab,kw OR (Intervention, Percutaneous Coronary):ti,ab,kw OR (Interventions, Percutaneous Coronary):ti,ab,kw OR (Percutaneous Coronary Revascularization):ti,ab,kw OR (Percutaneous Coronary Revascularizations):ti,ab,kw OR (Coronary Revascularization, Percutaneousy):ti,ab,kw OR (Revascularization, Percutaneous Coronary):ti,ab,kw OR (Revascularizations, Percutaneous Coronary):ti,ab,kw | 13521 |
| #2 | (Exercise):ti,ab,kw OR (Exercises):ti,ab,kw OR (Physical Activity):ti,ab,kw OR (physical exercise):ti,ab,kw OR (Training):ti,ab,kw | 242632 |
| #3 | (Trainings):ti,ab,kw OR (Motor Activity):ti,ab,kw OR (Tai Chi):ti,ab,kw OR (Vibration):ti,ab,kw OR (yoga):ti,ab,kw | 25672 |
| #4 | (wuqinxi):ti,ab,kw OR (baduanjin):ti,ab,kw OR (yijinjing):ti,ab,kw OR (kickboxing):ti,ab,kw OR (Pilates):ti,ab,kw | 1549 |
| #5 | (Balance):ti,ab,kw OR (Resistance):ti,ab,kw | 104658 |
| #6 | #5 OR #6 OR #7 OR #8 | 319960 |
| #7 | (Randomized controlled trial):ti,ab,kw OR (controlled clinical trial):ti,ab,kw OR (randomized):ti,ab,kw | 1185893 |
| #8 | #1 AND #6 AND #7 | 795 |

Supplementary Table 1. Search strategy of English database
